# Multi-segment osteotomy with interlocking intramedullary nail fixation in the treatment of lower limb deformity in older children with hypophosphatemic rickets

**DOI:** 10.3389/fped.2024.1332531

**Published:** 2024-02-19

**Authors:** Ming Lu, Haifeng You, Yukun Wang

**Affiliations:** Department of Pediatric Orthopedics, Beijing Jishuitan Hospital, Capital Medical University, Beijing, China

**Keywords:** osteotomy, interlocking intramedullary nail, hypophosphatemic rickets, older children, lower limb deformity

## Abstract

**Objective:**

Malformations of the lower limbs caused by hypophosphatemic rickets in older children are mostly complex, occurring on multiple planes without a single apex and showing arcuate bending of the diaphysis combined with torsion deformity, and are difficult to correct. This study retrospectively investigated the effect of and indicators for multi-segment osteotomy with interlocking intramedullary nail fixation in the treatment of bony deformity caused by hypophosphatemic rickets.

**Methods:**

The clinical data of 21 hypophosphatemic rickets patients seen between August 2007 and March 2022 were collected. The age range of the patients at the first surgery was 11 years and 1 month old to 15 years and 3 months old, with an average age of 12 years and 8 months. There were 6 males and 15 females. All patients had abnormal alignment of their lower limbs, with 32 limbs having varus deformity and 10 limbs having valgus deformity.

**Results:**

A total of 67 surgeries were performed across the 21 patients, including 24 cases of femoral osteotomy with antegrade intramedullary nail fixation, 6 cases of femoral osteotomy with retrograde intramedullary nail fixation, and 20 cases of tibial osteotomy with interlocking intramedullary nail fixation. A total of 34 limbs eventually underwent interlocking intramedullary nail fixation, 9 with genu valgum and 25 with genu varus. All 21 patients were followed up for a period of 14∼96 months, with an average of 42.6 months. The ends of the osteotomies achieved bony union in 4–9 months (average 6.8 months), after which normal weight-bearing walking could be resumed. No infection, vascular or neurological complications, or nonunion occurred. During postoperative follow-up, the alignment the lower limbs passed through zone 1 in 13 limbs, zone 2 in 12 limbs, and zone 3 in 5 limbs. The overall rate of an excellent effect was 83.3%.

**Conclusion:**

For lower limb deformity caused by hypophosphatemic rickets in older children, multi-segment osteotomy and strong fixation with interlocking intramedullary nails can achieve good correction outcomes.

## Introduction

Calcium and phosphorus levels play a very important regulatory role in normal bone metabolism. Hypophosphatemic rickets is a group of calcium deposition disorders characterized by low blood phosphorus caused by phosphorus reabsorption dysfunction in the proximal renal tubules that leads to increased phosphorus excretion by the kidneys. The main clinical manifestations of hypophosphatemic rickets are abnormal alignment of the lower limbs (severe genu varus or genu valgum and arcuate bending of long bones), short stature, dental abnormalities (such as periodontal abscess), and pain, with an incidence of approximately 1/20,000. The disease has high teratogenic and disability rates, which place a considerable burden on society and the families of patients (1).

The most common type of hypophosphatemic rickets is X-linked hypophosphatemia, caused by the mutation of the phosphorus regulatory gene homologous to endopeptidases on the X chromosome; other forms of the disease include those caused by autosomal inheritance of mutations and sporadic cases. The clinical manifestations include an insensitivity to the usual dose of vitamin D, resulting in continued manifestations of active rickets after 2–3 years old. Laboratory features of the disease include decreased blood phosphorus, increased urine phosphorus, and generally normal blood calcium. Orthopedic manifestations include progressive genu varus and/or genu valgum; arcuate bending and torsion deformity of the diaphysis may occur as the disease progresses, and thus older children and adolescent patients may present with a compound deformity across multiple planes without a single vertex. Paley et al. (2) believed that this deformity is caused by a gradual bending deformation of the bone under the action of long-term abnormal stress due to the insufficient mechanical strength of the bone due to rickets.

Albright et al. (3) first described vitamin D-resistant rickets in 1937, for which treatment mainly depended on large doses of vitamin D. In the 1950s and 1960s, some scholars published their experiences in the orthopedic treatment of hypophosphatemic rickets (4–6). Vitamin D and a brace were mainly used to prevent and control the progression of the deformity. If severe deformity occurred, an osteotomy and cast fixation was performed. With the extensive use of antibiotics, Sofield and Millar (7) published their 10-year follow-up results on multi-segment osteotomy and intramedullary fixation in 1959. The cases included osteogenesis imperfecta and hypophosphatemic rickets, and the results showed that the deformity was effectively corrected and maintained.

Because familial hypophosphatemic rickets is a rare disease, there are currently no standard treatment guidelines or expert consensus for the bony malformations it causes. The initial treatment of hypophosphatemic rickets should be medical. Rubinovitch (8) reported that mild deformities of less than 15 degrees can be spontaneously corrected with appropriate drug treatment. Surgical intervention should be considered when the effect of medical treatment is not satisfactory. Pedersen (6) reported that after the individual enters adulthood, a gradually worsening genu varus leads to an obvious swing gait, easy fatigue, and frequent low back pain and hip and knee joint pain. Therefore, it is necessary to provide timely alignment correction to minors. Currently, full-length x-rays of both lower limbs are used to assess alignment abnormalities of the lower limbs and to develop surgical plans.

Some author believed that patients with residual growth potential greater than 3 years had good results with growth modulation method. The effect is poor for patients with little growth potential and near puberty. Due to the long duration of the disease, patients at this age have formed multiple plane complex deformities without a single vertex. For the correction of such complex deformity, the fixation method should be used after osteotomy is still inconclusive. In this study we recruited older children and teenagers with delayed epiphyseal closure treated with multi-segment osteotomy and interlocking intramedullary nail fixation to correct complex deformities of the patients' lower limbs. There are no reports about the application of this method for correcting deformities in patients in this age group. This study retrospectively investigated the effect of and indications for multi-segment osteotomy and interlocking intramedullary nail fixation in the treatment of hypophosphatemic rickets with bony deformity.

## Materials and methods

### General information

The medical records of all patients with hypophosphatemic rickets admitted to our department from August 2007 to March 2022 were reviewed, and 21 patients were finally selected for this study. Inclusion criteria: ① At least one part of the long bone of the lower limbs was treated by osteotomy and fixation with intramedullary nail; ② patients were clearly diagnosed with hypophosphatemic rickets in the endocrine clinic ③ patients were treated with regular oral medication for more than one year, and the preoperative blood phosphorus value recovered to more than 1 mmol/L. Exclusion criteria: ① The residual growth potential is greater than 3 years when undergoing intramedullary nail fixation; ② The follow-up is less than 12 months. The age of patients when they obtaining a definite diagnosis ranged from 2 year 3 months to 8 years, with an average age of 4 years 10 months. Conventional oral drugs mainly include phosphate solution, vitamin D and calcium. Vitamin D was stopped one week before surgery, and was continued after recovery of non-weight-bearing flexion and extension activities 2 weeks after surgery. The ages of the patients when they first underwent intramedullay nail fixation surgery were between 11 years 1 month and 15 years 3 months, with an average age of 12 years 8 months. There were 6 male patients and 15 female patients. All patients had an abnormal alignment in both lower limbs: 32 limbs had varus deformity, and 10 limbs had valgus deformity.

### Radiological evaluation

**Figure 1:**
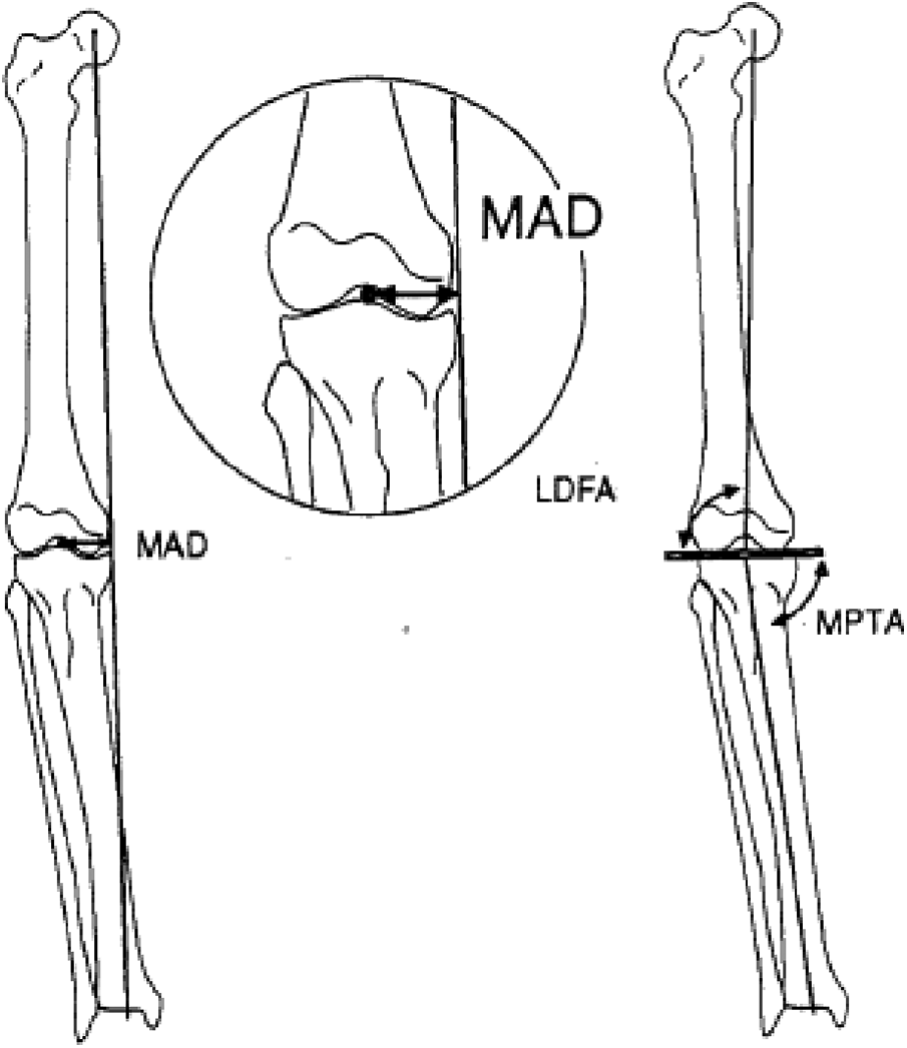
Measurement of the mLDFA, MPTA, and MAD values in the alignment for the lower limbs according to Paly's method (3).

Anteroposterior and lateral views of both knee joints and full-length radiographs of both lower limbs were taken before surgery. The full-length radiographs of both lower limbs were taken in the weight-bearing position with the patella located directly in front of the knee joint to eliminate errors caused by limb rotation. The mechanical lateral distal femoral angle (mLDFA), medial proximal tibial angle (mMPTA), mechanical axis deviation distance (MAD) between the mechanical axis and the center point of the knee joint, and the posterior inclination angle of the tibial plateau (Figure 1) were measured on the full-length anteroposterior radiographs of both lower limbs according to Paley's method (9). The patient's preoperative range of motion (ROM) was recorded, and the deformity angle at the center of rotation and angulation (CORA) was measured for deformity analysis and osteotomy planning. The diameter and length of the medullary cavity were measured, and appropriate intramedullary nails were selected.

### Surgical methods

Femoral and tibial intramedullary nails of the TRIGEN series from Smith & Nephew were used. Antegrade intramedullary nailing on the femur is described here as an example of the surgical procedures performed on the patients. First, the patient was placed in the supine position on the x-ray–permeable operating table; the patient was then positioned under a fluoroscope, and a 2–3 cm skin incision was made at the preoperatively planned CORA site to separate and expose the bone. Subperiosteal dissection and osteotomy were performed to remove a wedge-shaped bone fragment. Using a manual reamer, the bone was reverse reamed from the site of the osteotomy to the proximal femur, and the reamer broke through the cortex from the tip of the greater trochanter. Then, the manual reamer drill was used in an antegrade direction to ream from the first to the second osteotomy site planned before surgery. The skin and soft tissues were incised again with periosteum dissected. Another wedge-shaped bone piece was removed by osteotomy, and the drill continued to ream antegradely to the distal metaphysis. A guide wire was inserted through the opening of the greater trochanter. After proximal reaming was completed, the intramedullary nail was inserted along the guide wire. The interlocking screw was driven in at the proximal femur with a collimator; after that, the distal femur needs to be internally rotated when the osteotomy ends are aligned, and the distal locking is completed while maintaining the knee joint in a rotational neutral position to correct the external rotational deformity of distal femur. If the osteotomy site was still unstable, single-cortical locking plate technique was used to increase the local stability of the osteotomy site.

### Postoperative follow-up and evaluation

**Figure 2:**
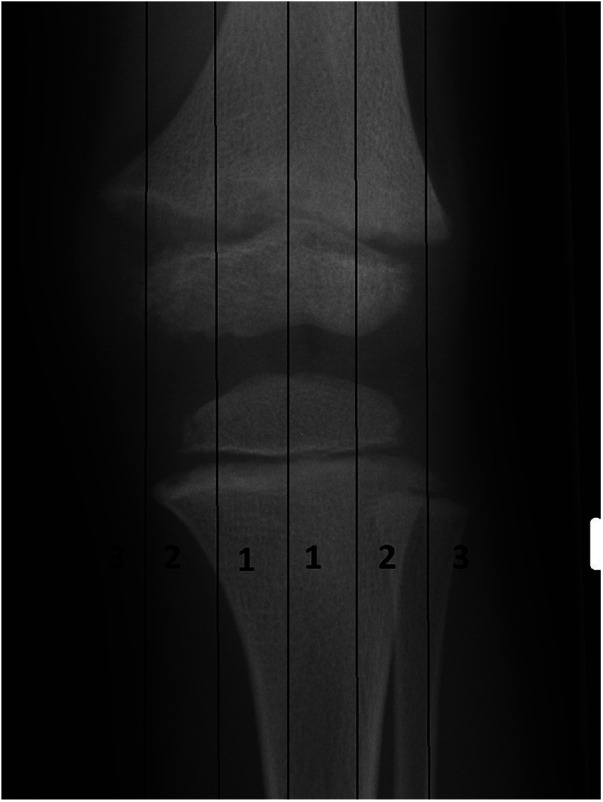
Stevens’ knee joint zoning. The knee joint is evenly divided into four parts: zone 1 is the medial and lateral central quarters, zone 2 is the medial and lateral edge quarters, and zone 3 is the outside edge of the knee joint.

Anteroposterior and lateral x-ray of the femur or tibia were taken at 6 weeks, 3 months, and half a year after surgery. Full-length anteroposterior and lateral x-ray of the lower limbs and knee joint were taken once a year after the osteotomy was healed. The mLDFA, mMPTA, MAD and the posterior inclination angle of the tibial plateau of the affected limb was measured on the full-length anteroposterior radiographs of lower limbs. The final correction was evaluated according to the knee joint zoning method described by Stevens (10) (Figure 2). If the mechanical alignment of the lower limb passed through the knee joint in zone 1, the effect of the surgery was considered excellent; if it passed through zone 2, it was considered good; and if it passed through zone 3, it was considered poor.

### Statistical methods

SPSS 17.0 software was used to analyze the normality of the data using the 1-sample Kolmogorov‒Smirnov test. The results showed that the distributions of the variables were not normal. Therefore, we used the Wilcoxon signed-rank test for nonparametric paired data to calculate the difference in mLDFA, MPTA, and MAD between the genu valgum group and the genu varus group before and after surgery.

## Results

**Figure 3:**
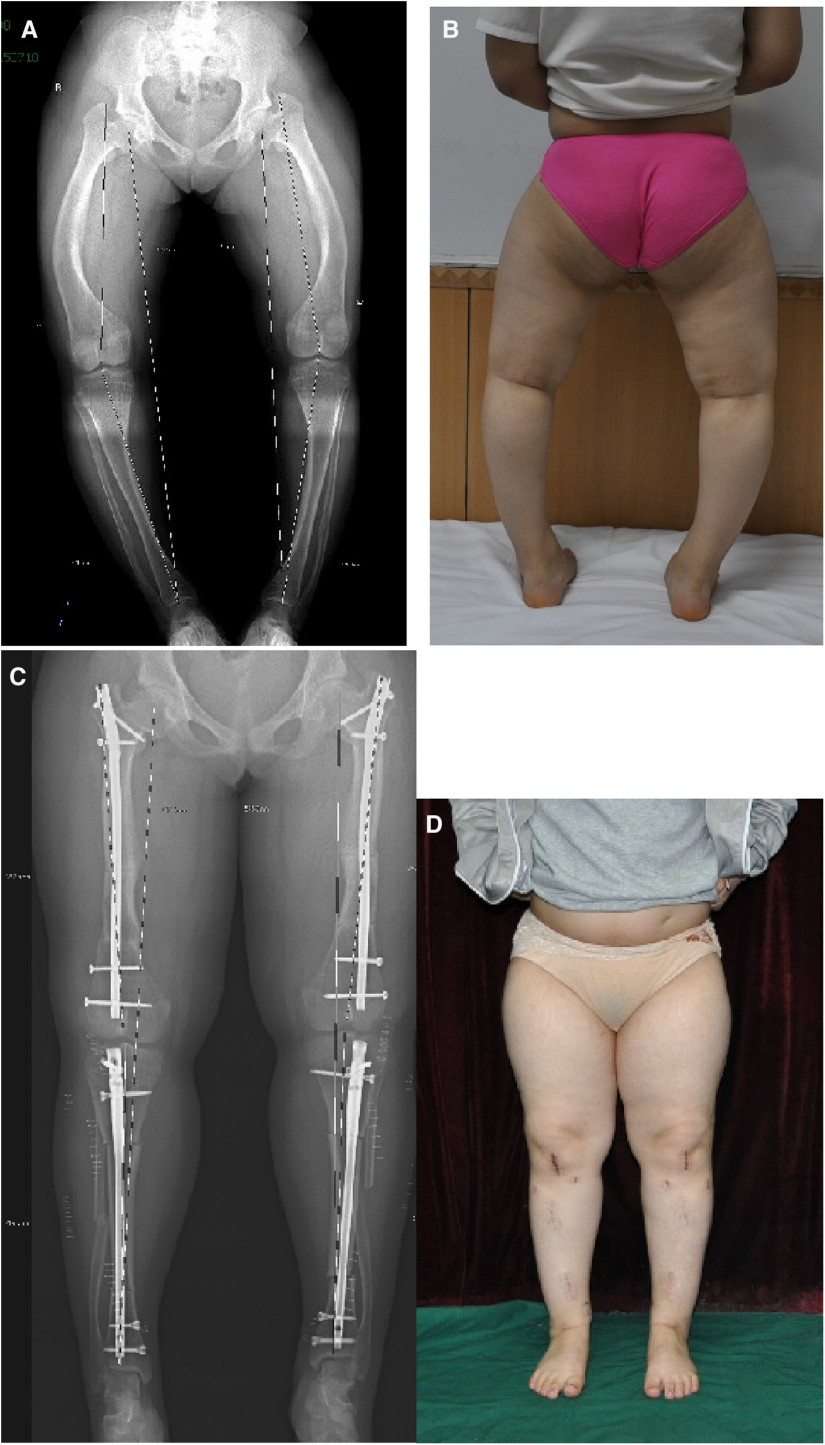
Typical case. **A** and **B** are the preoperative full-length radiograph and body image of the patient; **C** and **D** are the postoperative radiograph and body image of the patient.

The 21 patients included in this study received a total of 67 surgeries (Table 1), including 24 cases of femoral osteotomy with antegrade intramedullary nail fixation, 6 cases of femoral osteotomy with retrograde intramedullary nail fixation, and 20 cases of tibial interlocking intramedullary nail fixation; 6 cases of osteotomy with intramedullary nail with an additional unicortical locking plate to increase stability; 8 cases of tibial osteotomy with locking plate internal fixation, 6 cases of proximal tibial epiphysiodesis with eight plate, 1 case of distal femoral osteotomy with k-wire fixation, 1 case of interlocking screw removed, and 1 case of hollow screw fixation. Among a total of 50 cases of osteotomy and intramedullary nail fixation, 8 cases involved single femoral osteotomy alone, 1 case of single tibial osteotomy, and the rest underwent multi-segment osteotomy. Due to economic and family reasons, 6 patients only received surgical treatment on the most severe deformed side of the limb. A total of 34 limbs received interlocking intramedullary nail fixation, and 2 limbs were fixed by other methods. Of the limbs receiving intramedullary nail treatment, 9 limbs had genu valgum, and 25 limbs had genu varus. All 21 patients were followed up for 14–96 months (average 42.6 months). The osteotomy achieved clinical union in 4–9 months (average 6.8 months), and returned to normal weight-bearing walking. No infection, vascular or neurological complications, or nonunion occurred. All patients had good range of motion of the hip and knee joints before surgery and no pain; at the last follow-up after surgery, there was no restriction of motion of the hip and knee joints and no complaints of pain. Typical cases are shown in Figure 3.

**Table 1:** Patient general information and surgical treatment data.

No.	Sex	Age	Side	Alignment	Femur	Tibia	Combined surgery	Revision
1	Female	12	Left	Varus	Antegrade intramedullary nail	Locking plate		
			Right	Varus	Antegrade intramedullary nail	Locking plate		
2	Female	15	Right	Valgus	Retrograde intramedullary nail			
3	Female	11	Left	Varus		Intramedullary nail		
			Right	Varus		Intramedullary nail		Locking plate and eight plate
4	Female	13	Left	Varus		Intramedullary nail		locking plate
			Right	Varus		Intramedullary nail		locking plate
5	Male	13	Left	Varus	Antegrade intramedullary nail	Intramedullary nail	Internal fixation with hollow screw for slipped capital femoral epiphysis	
			Right	Varus	Antegrade intramedullary nail	Intramedullary nail		
6	Female	12	Left	Valgus	Retrograde intramedullary nail	Intramedullary nail		
			Right	Valgus	Retrograde intramedullary nail	Intramedullary nail	Nail removal	
7	Male	13	Left	Valgus	Retrograde intramedullary nail			
8	Female	14	Left	Varus	Antegrade intramedullary nail	Intramedullary nail		
			Right	Varus	Antegrade intramedullary nail	Intramedullary nail		
9	Female	12	Left	Varus	Retrograde intramedullary nail	Intramedullary nail		
			Right	Varus	Retrograde intramedullary nail	Intramedullary nail		
10	Female	12	Left	Varus	Antegrade intramedullary nail	Intramedullary nail	Removal of a previous eight plate	
			Right	Varus	Antegrade intramedullary nail	locking plate	Removal of the previous eight plate	
11	Male	15	Left	Varus	Antegrade intramedullary nail	Intramedullary nail		
			Right	Varus	Antegrade intramedullary nail	Intramedullary nail		
12	Female	13	Left	Varus	Antegrade intramedullary nail	Intramedullary nail		
			Right	Varus	Antegrade intramedullary nail	Intramedullary nail		
13	Female	11	Left	Varus	Antegrade intramedullary nail	Locking plate		
			Right	Varus		Locking plate		
14	Female		Right	Valgus	Antegrade intramedullary nail		Previous eight plate	
15	Male	14	Left	Valgus	Kirschner wire	Eight plate		
			Right	Varus	Antegrade intramedullary nail	Eight plate		
16	Male	11	Right	Varus	Antegrade intramedullary nail			
17	Female	13	Left	Varus	Antegrade intramedullary nail	Intramedullary nail		
18	Female	12	Left	Varus	Antegrade intramedullary nail	Intramedullary nail		
			Right	Varus	Antegrade intramedullary nail	Intramedullary nail		
19	Female	11	Left	Valgus	Antegrade intramedullary nail			
			Right	Valgus	Antegrade intramedullary nail			
20	Female	12	Left	Valgus	Antegrade intramedullary nail			
21	Male	13	Left	Varus	Antegrade intramedullary nail			
			Right	Valgus	Antegrade intramedullary nail			

In the genu valgum group, the average preoperative mLDFA was 70.6 degrees (65∼80 degrees), while the mLDFA at the final follow-up after surgery was 86.6 degrees (79–95 degrees); there was a significant difference (*P* = 0.021 < 0.05). The average MPTA preoperatively was 89.8 degrees (80∼102 degrees), while that at the final follow-up after surgery was 88 degrees (84–93 degrees); there was no significant difference. The preoperative MAD was 67.1 mm lateral (48–95 mm), and the MAD at the final follow-up after surgery was 8.4 mm (3∼28 mm) lateral; the difference was significant (*P* = 0.017 < 0.05).

In the genu varus group, the average preoperative mLDFA was 101.9 degrees (91–110 degrees), and the average mLDFA at the final follow-up after surgery was 95.1 degrees (83–104 degrees); the difference was significant (*P* < 0.001). The average preoperative MPTA was 77.4 degrees (66–86 degrees), and the average MPTA at the final follow-up after surgery was 85.2 degrees (74–95 degrees); the difference was significant (*P* = 0.001). The preoperative MAD was 77.4 mm medial (57–106 mm), and the postoperative MAD at final follow-up was 23.8 mm medial (6∼45 mm); the difference was significant (*P* < 0.001) (See Table 2).

**Table 2:** radiological outcomes of deformity correction

	Genu varus			Genu valgum		
	mLDFA (°)	MPTA (°)	MAD (mm)	mLDFA (°)	MPTA (°)	MAD (mm)
Preoperative	101.9	77.4	77.4	70.6	89.8	67.1
Final follow-up	95.1	85.2	23.8	86.6	88	8.4
P	<0.001	0.001	<0.001	0.021	>0.1	0.017

Significant deviations were observed in the preoperative alignment of the lower limbs in the 34 limbs that received intramedullary nail correction. At postoperative follow-up, the alignments were located in zone 1 for 13 limbs, 12 limbs in zone 2, and 5 limbs in zone 3. Three limbs had just completed osteotomy with intramedullary nail on the femoral side or the tibial side; their treatment is ongoing, and therefore, the final alignment of the lower limbs cannot be evaluated for the time being. One patient refused further surgery after the completion of the femoral side surgery. After surgery, the patient was only followed up with ordinary x-ray examinations and did not undergo full-length x-ray of the lower limb.

For 20 cases who underwent tibial interlocking intramedullary nail fixation, the average preoperative posterior inclination angle of the tibial plateau was 75.2 degrees (61–84 degrees), and the average postoperative posterior inclination angle of the tibial plateau was 74.4 degrees (61–81 degrees) at the final follow-up; there was no significant difference. In 3 cases, recurrence of proximal tibia deformity was observed at follow-up, and the interlocking intramedullary nail was removed, and proximal tibial osteotomy and locking plate fixation were performed for revision.

**Figure 4:**
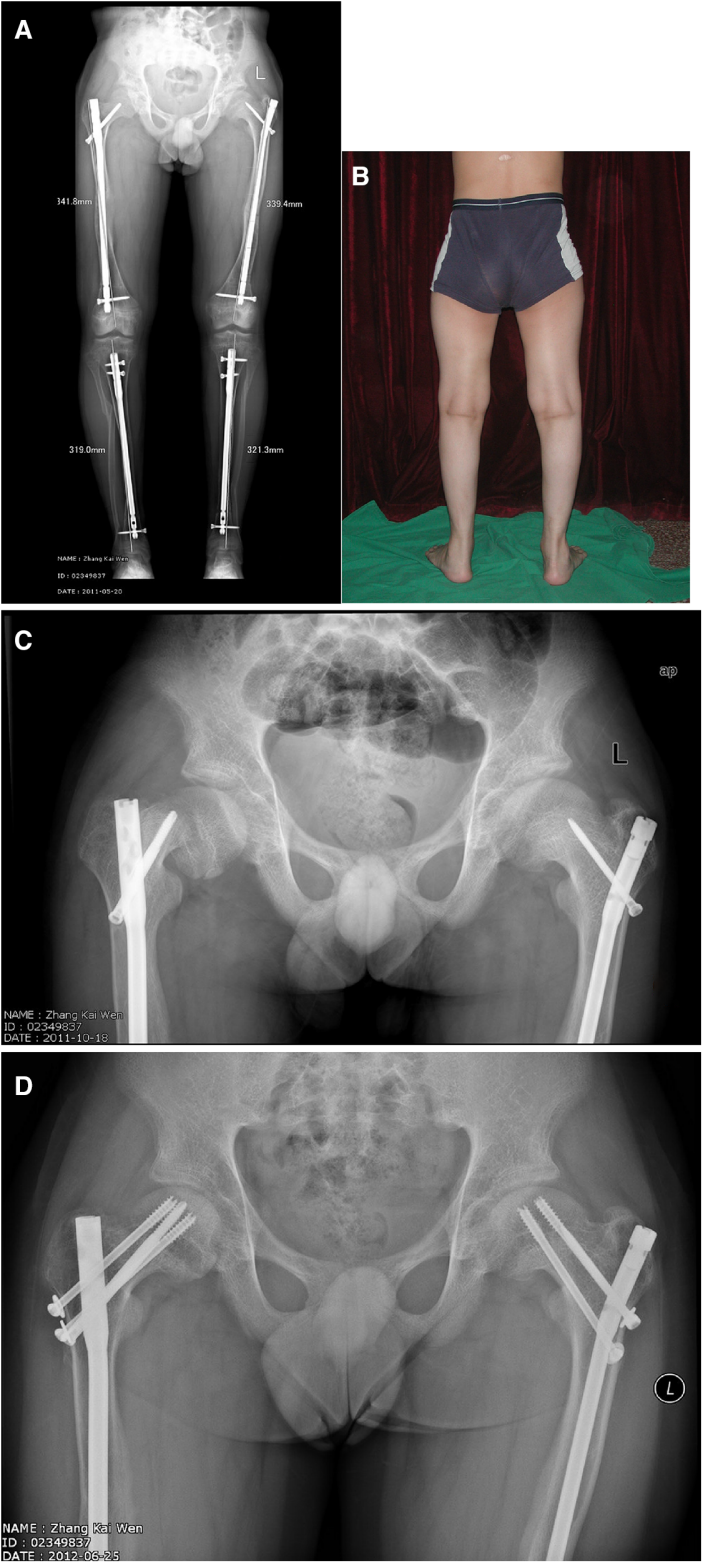
**A** and **B** are the follow-up of the patient one year after the completion of osteotomy and fixation; **C** shows a slipped capital femoral epiphysis happened in the right hip after minor trauma and exacerbation of coxa varus on the left side; **D**, reduction and fixation with longer interlocking screw and hollow screws.

For 30 cases who underwent intramedullary nail fixation of the femur, 1 case underwent removal of the interlocking screw due to concerns about growth arrest 5 months after retrograde intramedullary nail surgery. The osteotomy healed well at the follow-up 1 year after surgery, and the correction of the osteotomy on distal femur was partially lost at this time, but the deformity showed no progression at the 6-year follow-up after surgery. Another patient underwent bilateral femoral osteotomy with antegrade intramedullary locking nails. One year after surgery, the right femoral head had slipped from the epiphysis, and the left proximal femoral varus deformity worsened. Proximal interlocking screw replacement and hollow screw internal fixation were performed as reported by Li et al. (11) (Figure 4). The postoperative recovery was satisfactory, and the deformity of the proximal femur was not exacerbated during the 4-year follow-up.

## Discussion

The literature on the surgical treatment of children with familial hypophosphatemic rickets consists mostly of small case reports, among which the treatment methods used vary, and the evaluation criteria for surgical indications and postoperative follow-up are not clearly proposed. Stanitski (12) and Kanel (13) reported the use of an external fixator to correct deformities in hypophosphatemic rickets. The advantage of this method is that early weight-bearing is possible without immobilization, and thus, oral medication does not need to be adjusted. The complications included a few cases of needle tract infection and 1 case of fascial compartment syndrome. These two groups of patients were all followed up for a short time, and no recurrence was reported. Choi et al. (14) reported the application of the Ilizarov technique for osteotomy correction and simultaneous limb lengthening. The authors proposed that if the patient's serum phosphorus level is less than 2.5 mg/dl, bone lengthening during osteotomy is not recommended.

Stevens et al. (15, 16) proposed the application of guide growth in the treatment of bony malformations of familial hypophosphatemic rickets and achieved good outcomes. The patients in that study were all less than 10 years old. In 2017, Horn et al. (17) published the results of growth modulation with a eight plate in the treatment of bony deformity in hypophosphatemic rickets. The authors found that the efficacy of the growth modulation in patients with remaining growth potential of more than 3 years was significantly better than that of patients with less than 3 years of growth potential; the effects of guide growth for pubertal patients were poor.

Petje (18) reported the treatment results of a large number of osteotomies, approximately 98 cases. The authors found that in patients with recurrence after external fixation, the proportions of recurrence in the metaphysis and the diaphysis were the same, while 12 cases of intramedullary nails had recurrence only in the metaphysis at the distal and proximal ends. Song et al. (19) reported on 55 cases of surgical treatment and similarly found that among patients treated with an intramedullary nail, malformation recurrence only occurred in the metaphysis, while there was no recurrence of the malformation in the diaphysis. The authors advocated the use of elastic intramedullary nails and external fixators for osteotomy correction for young children with narrow medullary canals. An external fixator can correct multiplane complex deformity and lengthen the limb it at the same time, but the patient is prone to recurrence of the deformity and development of the lengthening site refracture, while intramedullary fixation with elastic nail alone has the problem of poor stability on osteotomy site; the combined use of the two can greatly enhance the fixation stability, maintain the correction effect and prevent refracture. Additionally, the combined procedure can reduce the use time of the external fixator and the probability of needle tract infection are lessened.

For adolescent and adult patients with closed epiphyses, the current consensus is that intramedullary fixation is the best method because it can effectively prevent the recurrence of the malformation (20). Osteotomy and intramedullary nail fixation assisted by external fixators has been promoted by scholars in recent years (21, 22). It has been reported that with this method, after intraoperative osteotomy and temporary fixation with an external fixator, the angle of the alignment of the lower limb can be precisely adjusted, and the interlocking intramedullary nail can be used to complete the final strong fixation and achieve good outcomes.

All patients in the group in the current study were older than 11 years and were of prepubertal age without epiphyseal closure. Patients with hypophosphatemic rickets are characterized by a short stature and low growth rate. According to Horn et al. (17), the residual growth potential of patients in this age group is insufficient to meet the needs of guide growth treatment. In addition, among patients of older age, the arcuate bending and torsion deformity of the dialysis cannot be solved by growth modulation. In this group of cases, to prevent further exacerbation of the deformity in the limb that was not treated with osteotomy, 6 patients temporarily underwent epiphysiodesis with a eight plate. The follow-up evaluation of these 6 patients only showed that the deformity did not exacerbate, but no correction was obtained, which further confirmed that guide growth is not a suitable deformity correction method for patients in this age group. In clinical practice, we found that the inner diameter of the medullary cavity in patients in this age group was close to that of adults and could accommodate the interlocking intramedullary nail after reaming. Therefore, we attempted to perform fixation with the interlocking intramedullary nail after osteotomy, achieving the goal of strong fixation and early exercise, and also reduce the chance of relapse.

When using interlocking intramedullary nail fixation for patients with an open epiphysis, the primary problem is avoiding interference with growth and development. For the femoral side, antegrade intramedullary nail fixation was mainly used. The proximal entrance was at the tip of the greater trochanter, and the distal end of the intramedullary nail terminated in the metaphysis. All interlocking screws were placed in the metaphysis without causing epiphysiodesis and did not affect femoral growth. Regarding the epiphysiodesis effect of the intramedullary nail passing through the epiphysis of the greater trochanter, we considered that the growth pattern of the greater trochanter epiphysis could be characterized as accumulative growth, accounting for a very small proportion of the development of femoral length and mainly affecting the morphological development of the proximal femur. After the epiphysiodesis, a coxa valga effect is produced; in contrast, the deformity of the proximal femur in hypophosphatemic rickets is coxa vara, which is helpful for the control and improvement of the proximal femoral deformity. No further aggravation of the proximal femoral deformity was found in our follow-up.

In this group of patients, 6 femurs underwent retrograde intramedullary nail fixation. We chose to insert the tail end of the intramedullary nail deep into the secondary ossification center of the distal femur to ensure that all the distal interlocking screws were all located in the metaphysis and avoid the epiphysiodesis. At the same time, the lengthened tail cap is used to flatten the end of the tail cap with the femoral intercondylar fossa cartilage, retaining the possibility of secondary removal. In addition, the intramedullary nail is not routinely removed after the osteotomy has healed. The retention of the nail can produce a space-occupying effect, avoid the formation of bone bridges, and affect length development. For the tibial intramedullary nail, because the entry point is located in the proximal metaphysis of the tibia, the main concern is that the genu recurvatum deformity could occur after the development of epiphysiodesis. We also used metaphyseal interlocking with an extended tail cap. In the postoperative follow-up, the posterior inclination of the tibial plateau was not significantly different from the preoperative level, and no deformity occurred.

The treatment outcome of patients was evaluated according to the knee joint zonal method of Stevens (10), and the final mechanical alignment of the lower limb was located in zones 1 and 2 for a total of 25 limbs, with an overall good rate of 83.3%. For patients with the final alignment of the lower limbs in zone 2, 8 limbs had insufficient correction of femoral varus, with an mLDFA greater than 93 degrees; 1 limb had insufficient correction of femoral valgus; and 3 patients had insufficient correction of the tibial side, with an MPTA less than 80 degrees. For patients with the final alignment of the lower limb in zone 3, the tibial side was insufficiently corrected in 4 limbs, and the femoral side was insufficiently corrected in 1 side.

The main characteristic of femoral side deformity is that the patient has deformity in both coronal plane and sagittal plane, and often combined with torsion deformity, so there are multiple deformity CORA points. Therefore, at least two sites should be selected in the design of the osteotomy procedure; in five of the eight cases with inadequate femoral side correction, only a single osteotomy was performed. Another reason for inadequate correction is that the preoperative anteroposterior and lateral x-ray projections can only reflect partial deformities, and it is difficult to accurately calculate the CORA point of the malformation; thus, it is difficult to precisely control the intraoperative osteotomy.

At the same time, the choice of antegrade or retrograde intramedullary nail fixation can also influence the effect of deformity correction. Due to the wide medullary cavity of the distal femur, the stability of antegrade intramedullary fixation in the distal osteotomy site of the femur is weakened, and intraoperative loss of correction is prone to occur. Most genu valgus deformities are mainly located at the distal femur, and therefore, for patients with severe deformities of genu valgum happened in distal femur, the use of retrograde intramedullary nails is recommended. Although the distal control of retrograde intramedullary nail has strong, genu varum patients often also present with arcuate bending deformity of the sagittal plane of the middle and upper segment of the femur. The working length of retrograde intramedullary nail for proximal fixation after multi-segment osteotomy is too short, and the stability is not good. So antegrade intramedullary nails should still be chosen for patients with larger arcuate bending of the middle and upper segments. For poor control of the distal deformity in correction of the distal femur, the future solution can refer to the experience of adult patients (20–22). After temporary fixation with an external fixator for precisely adjusting the alignment, the fixation is completed by the antegrade intramedullary nail fixation assists with the intramedullary blocking screw technique.

The main reason for the insufficient correction of tibial deformities is that in some patients, the deformity is very close to the proximal metaphysis of the tibia, and the intramedullary nail fixation needs to retain a certain length at the proximal to accommodate the interlocking screws in the metaphysis and control the bone fragments. The osteotomy cannot be completed at the true CORA level, and thus, the proximal tibial deformity is still retained during the surgery. During the follow-up, 3 patients experienced the deformity recurrence and worsened with weight-bearing after surgery. The lower limb alignment was shifted back to zone 3, so the intramedullary nail was removed, and proximal tibial osteotomy and locking plate fixation were performed for revision. A possible solution to avoid this situation is to keep the osteotomy line as close to the proximal tibia as possible. At this time, the problem of the wide medullary cavity of the proximal segment of the tibia and the poor stability of intramedullary fixation need to be addressed. During the insertion of the intramedullary nail, due to the relative osteoporosis of the patient itself, the nail is easy to swing and lead to the loss of correction. A possible solution is to use the suprapatellar approach for intramedullary nail implantation, which helps to control the angulation of the sagittal plane of the proximal bone fragment and facilitates intraoperative fluoroscopic monitoring. The addition of a single cortical locking plate or blocking nails can also help to increase fixation strength.

Proximal femoral deformities in hypophosphatemic rickets are mostly mild coxa vara. No patient in this group underwent subtrochanteric valgus osteotomy for coxa vara deformity, so conventional locking was typically performed by most surgeons in the direction from the greater trochanter to the lesser trochanter, and no worsening of the deformity of the proximal femur was found in postoperative follow-up. Reconstructional locking along the femoral neck were used in 3 patients. One patient developed a right slipped capital femoral epiphysis due to minor trauma one year after surgery, and the left coxa vara also worsened. Li et al. (11)reported that a similar situation can occur in children after internal fixation of femoral neck fractures. They believed that the causes of slipped femoral epiphyses are coxa vara caused by poor fracture reduction, the position of metal internal fixation, and premature weight-bearing. The reasons for the slipped epiphysis in this patient were mainly that the patient had coxa vara deformity, and the front of the interlocking screw in the reconstruction direction was located only in the metaphysis (Figure 4), resulting in the concentration of stress here. The rickets makes the weak metaphyseal cancellous bone region more fragile, so the epiphysis slipped under the action of weight-bearing varus scissor stress. Based on this situation, we do not recommend the routine use of the reconstruction direction for proximal locking. If the osteotomy site is near the proximal femur, and the reconstructive locking method must be used. The appropriate length should be selected so that the interlocking screw could passes through the physis plate and enters the secondary ossification center of proximal femur to complete the locking.

The limitation of this article is that the number of cases is insufficient to conduct a more detailed statistical analysis for the undercorrection cases to obtain more information that can help improve correction schedule. In future studies, full-length CT examinations of the lower limbs should be added to accurately evaluate torsional deformities of the lower limbs and formulate surgical correction plans. At the same time, preoperative and postoperative pelvic x-rays should be routinely taken to monitor the evolution of proximal femoral deformity.

## Summary

Older children and adolescents suffered hypophosphatemic rickets with open epiphyses mostly present with complex lower limb deformities in multiple planes without a single apex; treatment is currently difficult, and there is no consistently recommended treatment method. In this group of patients, multi-segment osteotomy and strong fixation with interlocking intramedullary nails were used, and good correction results were achieved, but there are certain limitations. Incomplete correction of the femoral deformity was the main cause of the postoperative alignment of the lower limb in zone 2; insufficient correction of the proximal tibial deformity was the main cause of the recurrence of postoperative tibial varus deformity. These problems remain to be further addressed by improving the intraoperative temporary fixation methods, precise control of osteotomy, and adding other assisted internal fixation.

## Data Availability

The raw data supporting the conclusions of this article will be made available by the authors, without undue reservation.
